# Supplementary material for: Explaining socioeconomic inequality in food consumption patterns among households with women of childbearing age in South Africa
Source: PLOS Glob Public Health. 2024 Oct 21;4(10):e0003859. doi: 10.1371/journal.pgph.0003859 (PMC11493276; doi:10.1371/journal.pgph.0003859)
Supplement: S1 Table — (DOCX) [file pgph.0003859.s001.docx]

**S1 Table. NOVA food classification system* based on the nature, extent and purpose of industrial food processing**

| **Food groups and definition**  **1 Unprocessed or minimally processed foods**  Unprocessed foods are those obtained directly from plants or animals (such as green leaves and fruits or eggs and milk) and purchased for consumption without undergoing any alteration following their removal from nature. Minimally processed foods are natural foods that have the cleaning, removal of inedible or unwanted parts, fractioning, grinding, drying, fermentation, pasteurisation, cooling, freezing, or other processes which do not add substances to the original food. The purpose of minimum processes is to preserve foods and make it possible to store them and, sometimes, also to decrease stages of food preparation (cleaning and removing inedible parts), facilitate their digestion, or render them more palatable (grinding or fermentation). | **Examples**  Fruits; vegetables; grains or cereals; potatoes and tubers; pulses (dried beans, peas and lentils); meat (beef, pork, mutton and other meat products) and fish; dairy; eggs; unsalted nuts and seeds; dried herbs, coffee and tea. |
| --- | --- |
| **2 Processed culinary ingredients**  These are substances extracted from natural foods or from nature itself by processes such as pressing, grinding, crushing, pulverising, and refining. The purpose of processing here is to obtain ingredients used in homes and restaurant kitchens to season and cook natural or minimally processed foods and to create with them varied and enjoyable dishes such as soups and broths, salads, rice and beans dishes, grilled or roasted vegetables and meat, and homemade bread, pies, cakes, and desserts. | Fats and oils; table sugar; flours; pasta; honey and table salt. |
| **3 Processed food products**  These are relatively simple products manufactured essentially with the addition of salt or sugar or other substances of common culinary use, such as oil or vinegar, to natural or minimally processed foods. The purpose here is to prolong the duration of foods and modify their palatability. | Tinned vegetables, legumes or fruits; salted nuts or seeds; processed meat or fish; tinned fish; cheese and bread. |
| **4** **Ultra-processed food products**  These are food and drink products whose manufacture involves several stages and various processing techniques and ingredients, many of which are used exclusively by industry. The purpose of processing here is to create durable, accessible, convenient, and highly palatable, ready-to-drink, ready-to-eat, or ready-to-heat products typically consumed as snacks or desserts or as fast meals which replace dishes prepared from scratch. | Confectionary (ice cream, chocolate, sweets); sugar-sweetened beverages, snacks; breakfast cereals; fast food, ready-to-eat and ready-to-heat meals; and baked goods. |

*Adapted from Monteiro et al. [1] and Moubarac et al. [2]

**References**

1. Monteiro C, Cannon G, Levy R, Claro R, Moubarac J, Martins A, et al. The Food System. Processing. The big issue for disease, good health, well-being. World Nutr. 2012;3(12):527–69.

2. Moubarac JC, Claro RM, Baraldi LG, Levy RB, Martins AP, Cannon G, et al. International differences in cost and consumption of ready-to-consume food and drink products: United Kingdom and Brazil, 2008-2009. Global public health. 2013;8(7):845-56. Epub 2013/06/06. doi: 10.1080/17441692.2013.796401. PubMed PMID: 23734735.
