# Supplementary material for: Explaining socioeconomic inequality in food consumption patterns among households with women of childbearing age in South Africa
Source: PLOS Glob Public Health. 2024 Oct 21;4(10):e0003859. doi: 10.1371/journal.pgph.0003859 (PMC11493276; doi:10.1371/journal.pgph.0003859)
Supplement: S2 Table — (DOCX) [file pgph.0003859.s002.docx]

**S2 Table. Descriptive Statistics of households with at least one woman aged 15 – 49 years in South Africa, 2005/06 and 2010/11**

| Variables | 2005/06 | 2010/11 |
| --- | --- | --- |
| Number of households | 21,144 | 25,328 |
| Proportion of households with at least one woman aged 15 - 49 years | 76.7 | 68.0 |
| Number of households with at least one woman aged 15 - 49 years | 16,209 | 17,217 |
| Female-headed household | 46.7%  (45.6 - 47.9) | 46.0  (45.1 -46.9) |
| **Socioeconomic quintile** |  |  |
| Q1 (poorest) | 17.1  (16.3 - 17.9) | 18.8%  (18.1 - 19.5) |
| Q2 | 17.7  (16.9 - 18.5) | 19.0  (18.3 - 19.8) |
| Q3 | 18.9  (18.1 - 19.7) | 19.0  (18.3 - 19.7) |
| Q4 | 20.5  (19.6 - 21.4) | 19.6  (18.9 - 20.4) |
| Q5 (richest) | 25.8  (24.8 - 26.9) | 23.5  (22.6 – 24.4) |
| **Population group** |  |  |
| Black African | 76.5  (75.4 - 77.5) | 77.9  (77.1 - 78.7) |
| Coloured | 8.9  (8.3 - 9.5) | 9.6  (9.1 10.1) |
| Asian/Indian | 2.7%  (2.3 - 3.1) | 2.9  (2.5 - 3.2) |
| White | 11.8  (10.9 - 12.8) | 9.7  (9.0 - 10.4) |
| **Area of residence** |  |  |
| Urban | 63.6  (62.6 - 64.6) | 66.5  (65.7 - 67.3) |
