# Supplementary material for: Explaining socioeconomic inequality in food consumption patterns among households with women of childbearing age in South Africa
Source: PLOS Glob Public Health. 2024 Oct 21;4(10):e0003859. doi: 10.1371/journal.pgph.0003859 (PMC11493276; doi:10.1371/journal.pgph.0003859)
Supplement: S3 Table — (DOCX) [file pgph.0003859.s003.docx]

**S3 Table. Decomposition of concentration index for spending of ultra-processed food products in South African households with women aged 15 to 49 years, 2005/06, 2010/11 and between the years**

|  | **2005/06** | | | **2010/11** | | | **Change** |
| --- | --- | --- | --- | --- | --- | --- | --- |
|  | **Elasticities**  **(i)** | **Concentration indices**  **(ii)** | **Contributions**  **(iii)** | **Elasticities**  **(iv)** | **Concentration indices**  **(iv)** | **Contributions**  **(v)** | **Contributions**  **vi = (v) – (iii)** |
| Female-headed household | -0.127***  (0.008) | 0.030  (0.024) | -0.004  (0.003) | -0.132***  (0.008) | 0.023  (0.020) | -0.003  (0.003) | 0.000  (0.000) |
| Socioeconomic status | 0.676***  (0.006) | 0.221***  (0.023) | 0.149***  (0.015) | 0.640***  (0.007) | 0.211***  (0.047) | 0.135***  (0.029) | -0.037**  (0.001) |
| **Population group** |  |  |  |  |  |  |  |
| Black African | -0.150***  (0.005) | -0.945***  (0.314) | 0.141***  (0.047) | -0.142***  (0.005) | -0.947***  (0.270) | 0.134***  (0.039) | 0.009**  (0.003) |
| Coloured | 0.108***  (0.020) | -0.041*  (0.023) | -0.004*  (0.002) | 0.132***  (0.016) | -0.051**  (0.022) | -0.007***  (0.003) | -0.004**  (0.000) |
| Asian/Indian | 0.469***  (0.038) | -0.009*  (0.005) | -0.004*  (0.002) | 0.545***  (0.029) | -0.019***  (0.005) | -0.010***  (0.002) | -0.007**  (0.000) |
| White | 0.808***  (0.007) | 0.013  (0.011) | 0.011  (0.009) | 0.789***  (-) | 0.000  (-) | 0.000  (-) | -0.030**  (0.001) |
| **Area of residence** |  |  |  |  |  |  |  |
| Urban | 0.184***  (0.005) | 0.211***  (0.011) | 0.039***  (0.002) | 0.154***  (0.005) | 0.134***  (0.021) | 0.021***  (0.003) | -0.018**  (0.000) |
| Residual |  |  | 0.170***  (0.036) |  |  | 0.161***  (0.012) | 0.015 |
| Total |  |  | 0.498***  (0.006) |  |  | 0.431***  (0.007) | -0.071 |

Significance levels are as follows: *** p< 0.01, ** p< 0.05, *p< 0.10. Bootstrapped standard error displayed in parentheses
