# Supplementary material for: Explaining socioeconomic inequality in food consumption patterns among households with women of childbearing age in South Africa
Source: PLOS Glob Public Health. 2024 Oct 21;4(10):e0003859. doi: 10.1371/journal.pgph.0003859 (PMC11493276; doi:10.1371/journal.pgph.0003859)
Supplement: Attachment — Submitted filename: Response letter PLOS GLobal Public Health-final.docx [file pgph.0003859.s004.docx]

24 February 2024

The Editor,

PLOS Global Public Health

**Fighting overweight and obesity in households with women of childbearing age through food consumption: explaining socioeconomic inequality in food consumption patterns in South Africa**

Dear Editor,

Thank you for your feedback. We have addressed the issues highlighted in italics and responded in bold (blue font).

1. *Please complete all items on the Human Participants Research Checklist that are relevant for your submission, by following this link: http://journals.plos.org/plosone/s/file?id=dc11/PLOSOne_Human_Subjects_Research_Checklist.docx. There may be overlap between the checklist items and other queries listed here; please address any duplicated queries both in your response email and on the checklist itself. Upload the completed Human Participants Research checklist as file type “Other” when you re-submit your manuscript. This document is for internal journal use only and will not be published if your article is accepted. The requested information will help us to assess whether your submission complies with PLOS’s policies and adheres to applicable reporting standards. Note that your manuscript may be rejected if you provide incomplete or inadequate responses to the checklist questions and that changing the ‘Section/Category’ of your article does not affect this requirement.*

**We have completed the checklist.**

2. *Your study includes live participants. Please include an Ethics Statement subsection to your Materials and Methods, to include:A statement that formal consent was obtained (including whether it was verbal or written) OR the reason consent was not obtained (e.g. anonymity)*

**This paper uses publicly available data that have received ethics approval. While this may need ethics approval, we still obtained this approval from the University of Cape Town’s Human Research Ethics Committee. As requested, the ethics approval document has been uploaded as part of this submission. We have also included details in the Ethics Statement subsection of the Methods.**

3. *Your study includes child participants, please update your Ethics Statement with confirmation that written informed consent was obtained from the parent/guardian of each participant under 18 years of age.*

**Please note that this study did not include child participants. So, there is no requirement to obtain any informed consent from parents/guardians.**

We have resolved the issues and resubmit the paper for your consideration to publish in the PLOS Global Public Health journal.

Yours sincerely,

Mweete Nglazi, on behalf of all co-authors
