# Supplementary material for: Explaining socioeconomic inequality in food consumption patterns among households with women of childbearing age in South Africa
Source: PLOS Glob Public Health. 2024 Oct 21;4(10):e0003859. doi: 10.1371/journal.pgph.0003859 (PMC11493276; doi:10.1371/journal.pgph.0003859)
Supplement: Attachment — Submitted filename: PGPH D 24 00301 March 24.docx [file pgph.0003859.s005.docx]

**Manuscript title:** Fighting overweight and obesity in households with women of childbearing age through food consumption: explaining socioeconomic inequality in food consumption patterns in South Africa

**Journal:** PLOS Global Public Health

**Manuscript ID:** PGPH-D-24-00301 – **March 2024**

**#1 Reviewer’s Report**

**Major revision**

**Major revision comments**

01. The title does not fit the manuscript. It suggests that this was an intervention study which is not. The study examines changes in socioeconomic inequalities in food consumption between two surveys and not any intervention to fight overweight and obesity.

02.The main analysis which used Oaxaca decomposition analyzed only UPF product spending which the authors claim is linked to obesity, yet the title mentions both overweight and obesity. Please explain this mismatch.

03. Who was overweight and obese in the study? No definition for this classification has been provided.

04. The authors need to explain and explore the assumption of homogeneity in 5 food consumption patterns among individuals within each household. Although the study only included women aged 15 to 49, most of the food consumption indices are calculated using the household size. Food consumption patter may be different for sex (male vs female) and age, for example infants <6 months should be exclusively breastfed.

05. Table 1 show the major key variables. Most of these variables have not been defined or explained, for example how was rural and urban residence defined? How were the socioeconomic quintiles calculated? Which household assets were included?

**#2 Reviewer’s Report**

**Minor revision**

The manuscript addresses a major public health issue affecting South African population. this is a very useful well conducted study that will go along way in assisting with formulation of workable strategies to curb overweight and obesity in South Africa and beyond. I have a few minor correction that need attention to improving the quality of the manuscript.

**Minor revision comments**

01. Pg 5 line 6. The acronym WCBA needs to be explained further.

02. Pg 11 line 15 and elswhere, the use of the abbreviation "i.e." should be discouraged and use the word itself.

03. Page 27 line 1 and elsewhere, organizations such as WHO should include their location when referencing.

03. Pg 27 line 42 and elsewhere, be consistent when writing journal names in the reference list by capitalising first letter in each word of the name or not doing it at all.

**Editor**

**Recommendation:** **Major revision**

**Main review questions**

**1. Originality:**

- Does the paper contain new information adequate to justify publication? Yes

**2. Introduction:**

- Does the paper clearly explain why the study was necessary? Yes

- Is the research question clear and appropriate? Yes

**3. Relationship to literature:**

- Does the paper fit the scope of the journal? Yes

- Does the paper adequately understand the relevant literature in the field? No

- Does the paper cite an appropriate range of literature sources? NO

**4. Theory, Data and Methodology:**

- Is the paper's argument built on an appropriate base of theory? No

- Does the paper utilize an appropriate database? Yes

- Is there enough detail to repeat the study? No

- Are the methods of analysis employed appropriate? Yes

**5. Results:**

- Are the results presented clearly and accurately? No

- Do the results presented match the methods? No

- Do the authors logically explain the findings? No

**6. Discussion and Conclusions:**

- Do the authors compare the findings with current findings in the research field? No

- Are the implications of the findings for future research and public policy discussed? No

- Are any contradictory data discussed? Yes

- Are any limitations of the study discussed? Yes

- Are the conclusions supported by the data presented? No

**7. Organisation and presentation:**

- Is the writing style clear and appropriate to the readership? Yes

- Is the paper well organized? Yes

- Does the content of the paper justify its length? Yes

**Revision comments**

01. Rewrite the title.

This is a study about socioeconomic inequality in food consumption.

02. Rewrite abstract.

Do not explain the meaning of standard statistical methods in the abstract.

Explain better the results. The text is unclear.

03. Delete the sentence “NOVA this is not an acronym”.

04. Put the ethics statement at the end of the paper.

05. There is no data on obesity.

Rewrite the paper by focusing on socioeconomic inequality in food consumption.

06. Changes in eating habits due to changes in disposable income are a well-known phenomenon (i.e., the so-called generalized Engel’s Law). This literature should be included in the Discussion section. For instance, Nsabimana et al. (2020).

07. Data should be available to other researchers upon request or stored in public databases.

Similar studies not cited in the paper:

Frank, T., Ng, S. W., Lowery, C. M., Thow, A.-M., & Swart, E. C. (2024). Dietary intake of low-income adults in South Africa: ultra-processed food consumption a cause for concern. Public Health Nutrition, 27(1). https://doi.org/10.1017/s1368980023002811

References

Nsabimana, A., Bali Swain, R., Surry, Y., & Ngabitsinze, J. C. (2020). Income and food Engel curves in Rwanda: a household microdata analysis. Agricultural and Food Economics, 8(1). https://doi.org/10.1186/s40100-020-00154-4
