# Supplementary material for: Explaining socioeconomic inequality in food consumption patterns among households with women of childbearing age in South Africa
Source: PLOS Glob Public Health. 2024 Oct 21;4(10):e0003859. doi: 10.1371/journal.pgph.0003859 (PMC11493276; doi:10.1371/journal.pgph.0003859)
Supplement: Attachment — Submitted filename: Response to reviewers.docx [file pgph.0003859.s006.docx]

**Manuscript title:** Fighting overweight and obesity in households with women of childbearing age through food consumption: explaining socioeconomic inequality in food consumption patterns in South Africa

**Journal:** PLOS Global Public Health

**Manuscript ID:** PGPH-D-24-00301 – **March 2024**

Dear Editor,

We would like to thank the reviewers for their helpful comments to strengthen the above captioned manuscript. Please find below a point-by-point response to the reviewers comments.

**#1 Reviewer’s Report**

**Major revision**

**Major revision comments**

01. The title does not fit the manuscript. It suggests that this was an intervention study which is not. The study examines changes in socioeconomic inequalities in food consumption between two surveys and not any intervention to fight overweight and obesity.

**Thank you for commenting on our manuscript. The title has been rewritten as follows: “*Explaining socioeconomic inequality in food consumption patterns among households with women of childbearing age in South***

02.The main analysis which used Oaxaca decomposition analyzed only UPF product spending which the authors claim is linked to obesity, yet the title mentions both overweight and obesity. Please explain this mismatch.

**As initially suggested by the reviewer, we have amended the title, removing overweight and obesity. It was rewritten by focusing on socioeconomic inequality in food consumption, the paper’s main focus. We believe that this deals with the potential mismatch raised by the reviewer.**

03. Who was overweight and obese in the study? No definition for this classification has been provided.

**Apparently, this comment was due to the introduction of obesity and overweight in the title. The reviewer is right. In the revised manuscript, overweight and obesity have been removed from the title and are not directly relevant to the manuscript. We apologise for the confusion this may have caused.**

04. The authors need to explain and explore the assumption of homogeneity in food consumption patterns among individuals within each household. Although the study only included women aged 15 to 49, most of the food consumption indices are calculated using the household size. Food consumption patter may be different for sex (male vs female) and age, for example infants <6 months should be exclusively breastfed.

**We agree with the reviewer regarding the assumption of homogeneity in food consumption at the household level. In fact, we noted this issue in our limitations as we do not have the individual-level data needed to tease out this complexity. While we think that our assumption was reasonable, given our focus on adults, we agree that further analysis with individual-level data will provide more insight. Please see our revision in the limitations section:**

**“While this alignment may not always be the case, as consumption patterns may differ by age and sex, there is likely a substantial correlation between individual and household consumption patterns for a large portion of the population, more so for the less wealthy. We note that further research is needed using data at the individual level that focuses on women of childbearing age 15 – 49 years to address the assumption of homogeneity in food consumption patterns among individuals within each household.”**

05. Table 1 show the major key variables. Most of these variables have not been defined or explained, for example how was rural and urban residence defined? How were the socioeconomic quintiles calculated? Which household assets were included?

**We have now edited Table 1 as the reviewer requested by adding notes to Table 1. Please see the following notes:**

**“Urban residence refers to households in a big city (metro), town or township, while rural residence refers to rural or traditional areas. Enumerators were trained to record the location of each household; Quintiles of socioeconomic status, which groups the population into five quintiles, each containing approximately 20% of the population, are based on household consumption expenditure per capita.”**

**Also, an explanation of the socioeconomic status measure was added to the main manuscript. Please note that we did not use the asset index; as such, there are no assets to be listed.**

**“Consumption expenditure per capita was used to measure socioeconomic status, which was obtained by dividing total consumption expenditure at the household level by the total household size.”**

**#2 Reviewer’s Report**

**Minor revision**

The manuscript addresses a major public health issue affecting South African population. this is a very useful well conducted study that will go along way in assisting with formulation of workable strategies to curb overweight and obesity in South Africa and beyond. I have a few minor correction that need attention to improving the quality of the manuscript.

**Thank you for your interest in our manuscript and for your helpful comments on improving its quality.**

**Minor revision comments**

01. Pg 5 line 6. The acronym WCBA needs to be explained further.

**Thank you for pointing this out. We have explained that WCBA means women of childbrearing age in the revised manuscript.**

02. Pg 11 line 15 and elswhere, the use of the abbreviation “i.e.” should be discouraged and use the word itself.

**We have changed all occurrences of i.e. into that is, as suggested.**

03. Page 27 line 1 and elsewhere, organizations such as WHO should include their location when referencing.

**Thank you. The location is included when referencing organizations as recommended.**

03. Pg 27 line 42 and elsewhere, be consistent when writing journal names in the reference list by capitalising first letter in each word of the name or not doing it at all.

**Thank you. The referencing is consistent when writing the journal names.**

**Editor**

**Recommendation:** **Major revision**

**Main review questions**

**1. Originality:**

- Does the paper contain new information adequate to justify publication? Yes

**2. Introduction:**

- Does the paper clearly explain why the study was necessary? Yes

- Is the research question clear and appropriate? Yes

**3. Relationship to literature:**

- Does the paper fit the scope of the journal? Yes

- Does the paper adequately understand the relevant literature in the field? No

- Does the paper cite an appropriate range of literature sources? NO

**4. Theory, Data and Methodology:**

- Is the paper’s argument built on an appropriate base of theory? No

- Does the paper utilize an appropriate database? Yes

- Is there enough detail to repeat the study? No

- Are the methods of analysis employed appropriate? Yes

**5. Results:**

- Are the results presented clearly and accurately? No

- Do the results presented match the methods? No

- Do the authors logically explain the findings? No

**6. Discussion and Conclusions:**

- Do the authors compare the findings with current findings in the research field? No

- Are the implications of the findings for future research and public policy discussed? No

- Are any contradictory data discussed? Yes

- Are any limitations of the study discussed? Yes

- Are the conclusions supported by the data presented? No

**7. Organisation and presentation:**

- Is the writing style clear and appropriate to the readership? Yes

- Is the paper well organized? Yes

- Does the content of the paper justify its length? Yes

**Revision comments**

01. Rewrite the title.

This is a study about socioeconomic inequality in food consumption.

**We agree with the Editor and the reviewers on this point. As a result, the title has been rewritten as follows:** **“Explaining socioeconomic inequality in food consumption patterns among households with women of childbearing age in South Africa.” We believe that this now reflects the contents of the paper better.**

02. Rewrite abstract.

Do not explain the meaning of standard statistical methods in the abstract.

Explain better the results. The text is unclear.

**The abstract was rewritten as suggested.**

03. Delete the sentence “NOVA this is not an acronym”.

**As suggested, the sentence “NOVA this is not an acronym” has been deleted.**

04. Put the ethics statement at the end of the paper.

**The ethics statement has been moved to the end of the paper.**

05. There is no data on obesity.

Rewrite the paper by focusing on socioeconomic inequality in food consumption.

**We rewrote the paper with focus on socioeconomic inequalitiy in food consumption.**

06. Changes in eating habits due to changes in disposable income are a well-known phenomenon (i.e., the so-called generalized Engel’s Law). This literature should be included in the Discussion section. For instance, Nsabimana et al. (2020).

**Thank you for this important point. We have added the literature to the discussion.**

07. Data should be available to other researchers upon request or stored in public databases.

**This is well noted. The dataset used for this work are available at the University of Cape Town’s DataFirst portal (**[**https://www.datafirst.uct.ac.za/**](https://www.datafirst.uct.ac.za/)**).**

Similar studies not cited in the paper:

Frank, T., Ng, S. W., Lowery, C. M., Thow, A.-M., & Swart, E. C. (2024). Dietary intake of low-income adults in South Africa: ultra-processed food consumption a cause for concern. Public Health Nutrition, 27(1). https://doi.org/10.1017/s1368980023002811

**We thank you for the useful paper, which was published very recently. We have included this in the paper.**

References

Nsabimana, A., Bali Swain, R., Surry, Y., & Ngabitsinze, J. C. (2020). Income and food Engel curves in Rwanda: a household microdata analysis. Agricultural and Food Economics, 8(1). https://doi.org/10.1186/s40100-020-00154-4

**We thank you for the useful paper. We have included this in the paper.**

We thank the Editor and Reviewers for helping us revise and strengthen this manuscript. We hope this revised version is acceptable for publication in the PLOS Global Public Health Journal.

Yours sincerely,

Mweete D. Nglazi, on behalf of all the authors
